# Influence of resuscitation status on the performance of APACHE III, APACHE IV and SAPS III

**DOI:** 10.1186/cc9925

**Published:** 2011-03-11

**Authors:** MT Keegan, O Gajic, B Afessa

**Affiliations:** 1 Mayo Clinic, Rochester, MN, USA

## Introduction

The presence of a do-not-resuscitate (DNR) order is an independent predictor of mortality in ICU patients [1]. Of the major ICU prognostic scoring systems, only MPM III includes DNR status as a predictor. The influence of DNR status on APACHE III and IV and SAPS III is unknown. We hypothesized that there would be differences in the performances of APACHE III, APACHE IV, and SAPS III when DNR status was included as a predictor variable.

## Methods

A retrospective cohort study was performed. Demographic, physiologic and outcome data for 2,596 patients admitted to one of three ICUs (medical, surgical, mixed) at our tertiary referral center in 2006 were collected. The presence or absence of a DNR order on ICU admission and at the end of the first ICU day was recorded. The performance of each of the four models, with and without inclusion of first-day DNR status, was assessed using the area under the receiver operating characteristic curve (AUC) for discrimination and the Hosmer-Lemeshow statistic (HLS) for calibration. Comparison of model performance was as described by Hanley [2].

## Results

Of the 2,596 patients studied, 211 (8.1%) and 252 (9.7%) had DNR orders on ICU admission and at the end of the first ICU day, respectively. Two hundred and eighty-three patients (10.9%) did not survive to hospital discharge. A total 19.4% of the nonsurvivors had DNR orders on admission versus 6.7% of the survivors, *P *< 0.01. At the end of the first ICU day, 32.5% of nonsurvivors were DNR versus 6.9% of survivors, *P *< 0.01. The AUCs (95% CI) of the models for prediction of hospital mortality were 0.868 (0.854 to 0.880), 0.861 (0.847 to 0.874) and 0.801 (0.785 to 0.816) for APACHE III and IV, and SAPS III, respectively. When DNR status at end of the first ICU day was included in the models, the AUCs were 0.876 (0.855 to 0.897), 0.868 (0.846 to 0.891), and 0.816 (0.791 to 0.841), respectively. There were no significant differences between the discriminative ability of the models with and without DNR status (APACHE III *P *= 0.103, APACHE IV *P *= 0.145, SAPS III *P *= 0.072). The HLS for the models with and without DNR status were 33.7 and 29.3, 31.0 and 33.3, and 36.6 and 29.0 for APACHE III and IV and SAPS III, respectively. Each of the HLS generated *P *< 0.05.

## Conclusions

Neither the discrimination nor calibration of APACHE III and IV and SAPS III were significantly improved by the inclusion of resuscitation status at the end of the first ICU day in the prognostic models.
